# Patients with community acquired pneumonia admitted to European intensive care units: an epidemiological survey of the GenOSept cohort

**DOI:** 10.1186/cc13812

**Published:** 2014-04-01

**Authors:** Andrew P Walden, Geraldine M Clarke, Stuart McKechnie, Paula Hutton, Anthony C Gordon, Jordi Rello, Jean-Daniel Chiche, Frank Stueber, Christopher S Garrard, Charles J Hinds

**Affiliations:** 1 Intensive care unit, Royal Berkshire hospital, Reading RG1 5AN, UK; 2 Wellcome Trust Centre for Human Genetics, University of Oxford, Oxford, UK; 3 Intensive care unit, John Radcliffe hospital, Oxford, UK; 4 Anaesthetics, Pain Medicine and Intensive care, Imperial College, London, UK; 5 Vall d’Hebron Univeristy hospital, Ciberes, Spain; 6 Hospital Cochin, Paris, France; 7 Anaesthesia, Bern University hospital and University of Bern, Bern, Switzerland; 8 William Harvey Research Institute, Queen Mary University of London, London EC1M 6BQ, UK

## Abstract

**Introduction:**

Community acquired pneumonia (CAP) is the most common infectious reason for admission to the Intensive Care Unit (ICU). The GenOSept study was designed to determine genetic influences on sepsis outcome. Phenotypic data was recorded using a robust clinical database allowing a contemporary analysis of the clinical characteristics, microbiology, outcomes and independent risk factors in patients with severe CAP admitted to ICUs across Europe.

**Methods:**

Kaplan-Meier analysis was used to determine mortality rates. A Cox Proportional Hazards (PH) model was used to identify variables independently associated with 28-day and six-month mortality.

**Results:**

Data from 1166 patients admitted to 102 centres across 17 countries was extracted. Median age was 64 years, 62% were male. Mortality rate at 28 days was 17%, rising to 27% at six months. Streptococcus pneumoniae was the commonest organism isolated (28% of cases) with no organism identified in 36%. Independent risk factors associated with an increased risk of death at six months included APACHE II score (hazard ratio, HR, 1.03; confidence interval, CI, 1.01-1.05), bilateral pulmonary infiltrates (HR1.44; CI 1.11-1.87) and ventilator support (HR 3.04; CI 1.64-5.62). Haematocrit, pH and urine volume on day one were all associated with a worse outcome.

**Conclusions:**

The mortality rate in patients with severe CAP admitted to European ICUs was 27% at six months. *Streptococcus pneumoniae* was the commonest organism isolated. In many cases the infecting organism was not identified. Ventilator support, the presence of diffuse pulmonary infiltrates, lower haematocrit, urine volume and pH on admission were independent predictors of a worse outcome.

## Introduction

Community acquired pneumonia (CAP) is common, affecting between 5 and 11 individuals per 1,000 of the adult population each year [1,2] and is the commonest cause of sepsis, severe sepsis and septic shock [3]. Between 22 and 42% of patients require hospital admission [1,2,4] of whom 5 to 10% will be admitted to an ICU [5-7]. Hospital and ICU admission rates for CAP are increasing for all ages [8]. An ageing, more vulnerable population, earlier recognition of deteriorating patients and better availability and use of intensive care beds may in part explain this increase.

A number of investigators have examined the clinical and microbiological factors that might affect the outcome from severe CAP [5-7,9-23], but these studies have often been relatively small, with considerable heterogeneity in both the inclusion criteria and outcome measures. As a consequence reported mortality rates have varied and there has been uncertainty regarding the most important risk factors for death. A number of more recent, larger studies have focussed on identifying patients with CAP at increased risk of severe sepsis and death, as well as those who may require ventilator or vasopressor support [3,24-26]. One of these studies provides outcome data at 90 days for the smaller subgroup of patients with severe sepsis admitted to the ICU [3], and another [24] reports microbiological findings and 90-day mortality in a subgroup of 170 patients admitted to the ICU.

The aim of the study reported here was to define the clinical characteristics, microbiological aetiology, outcomes and independent risk factors for mortality in a large, contemporary cohort of patients with severe CAP admitted to ICUs across Europe. By using such a large database with clear censor data for mortality we hoped to overcome some of the deficiencies of previous studies.

## Materials and methods

The GenOSept study was conceived by the European Critical Care Research Network (ECCRN), the research arm of the European Society for Intensive Care Medicine (ESICM) with the aim of defining genetic influences on the host response and outcomes in patients with sepsis. GenOSept is a pan-European study that has recorded comprehensive phenotypic data and obtained DNA from a large cohort of patients admitted to ICU with sepsis due to CAP, peritonitis, meningococcal disease or pancreatitis. Ethics approval was granted nationally, for individual centres, or for both. Written, informed consent was obtained from all patients or a legal representative. Patients reported here were recruited into GenOSept from 102 centres across 17 countries (see Additional file 1 for contributors) over a 4-year period between September 2005 and October 2009.

Inclusion criteria were as follows: admission to a high dependency unit (HDU) or ICU with CAP, and age over 18 years. The diagnosis of CAP was defined as a febrile illness associated with cough, sputum production, breathlessness, leucocytosis and radiological features of pneumonia acquired in the community or within less than 2 days of hospital admission [24]. The diagnosis of sepsis was based upon the International Consensus Criteria published in 2003 [27]. Patients were further sub-classified according to the criteria for severe sepsis and septic shock.

Exclusion criteria were as follows: patient or legal representative unwilling or unable to give consent; patient under 18 yrs of age; patient pregnant; advanced directive to withhold or withdraw life-sustaining treatment or admitted for palliative care only (please see also Additional file 1).

Patients were observed until death or for a maximum of 6 months. In those who had died between ICU discharge and 6-month follow-up the date of death was recorded. Specific data was recorded in the electronic case report form (eCRF) to allow calculation of the acute physiology and chronic health evaluation II (APACHE II) and sequential organ failure assessment (SOFA) scores [28,29]. The Infectious Diseases Society of America/American Thoracic Society (IDSA/ATS) criteria were used for the diagnosis of severe CAP [30]. Co-morbidities were recorded according to the modified Charlson scoring system [31]. Chest radiograph appearances were recorded as: 1) lobar, 2) localised or 3) diffuse bilateral. Investigators were also asked to consider the differential diagnosis of cardiogenic pulmonary oedema in those with diffuse pulmonary infiltrates. Microbiological investigations were performed according to local policies and practices. Investigators recorded the microbiological findings, including the organism(s) isolated, the source of the organism and the use of serology.

### Statistical methods

Mortality at 28 days and at 6 months were chosen as primary endpoints. Univariate analysis was performed using a Cox proportional hazards model, adjusted for age and gender. Variables considered by the investigators to be clinically relevant were chosen for analysis (see Additional file 1). A test for proportional hazards using the Schoenfeld residuals was performed, and for covariates indicating evidence of non-proportionality, spline smooth estimates of time-dependent hazard ratios (HRs) with point-wise confidence bands were calculated [32]. Variables that were significant in the univariate analysis after Bonferroni adjustment for multiple testing (*P*-value <0.05/*k* where *k* is the number of variables tested) were entered into a multivariate Cox proportional hazards model to determine independent risk factors for death. A final set of predictors for each of 28-day and 6-month mortality was selected from these variables via a stepwise Cox proportional hazards regression model. R software version 2.11.1 was used for all data analysis.

## Results

Between 29 September 2005 and 13 October 2009, 1,170 patients were enrolled. Four individuals were excluded because of missing or inconsistent data. Patient characteristics are shown in Table 1. On admission, 1,135 patients (97%) met IDSA/ATS criteria for severe CAP, 991 on major criteria and 146 on minor criteria.

**Table 1:** Demographic data for patients with CAP admitted to adult ICUs across Europe

**Case mix**	**Number analysed**	**Number**	**(%)**
Age	1,166		
<24 years		23	2
24 to <34 years		54	4.6
34 to <44 years		118	10.1
44 to <54 years		146	12.5
54 to <64 years		237	20.3
64 to <74 years		286	24.5
74 to <84 years		247	21.2
≥84 years		55	4.7
Gender	1,166		
Male		722	61.9
Female		444	38.1
Race	1,152		
African		17	1.5
Asian		11	1
Caucasian		940	81.6
Hispanic		34	3
Indian		3	0.3
Mediterranean		147	12.8
Country	1,166		
Belgium		147	12.6
Czech Republic		40	3.4
Germany		35	3
Estonia		17	1.5
Spain		194	16.6
France		97	8.3
Greece		6	0.5
Croatia		4	0.3
Ireland		21	1.8
Israel		5	0.4
Italy		51	4.4
Netherlands		2	0.2
Poland		50	4.3
Serbia		2	0.2
United Kingdom		495	42.5
Medical co-morbidities			
Heart and vascular disease	1,164	458	39.3
Respiratory disease	1,163	531	45.7
Neurological disease	1,164	153	13.1
Severe renal disease	1,134	79	7
Gastrointestinal disease	1,164	139	11.9
Malignancy	1,164	108	9.3
Diabetes	1,164	228	19.6
Other illness	1,164	145	12.5
Days in ICU	1,166		
<3		103	8.8
3 to <7		286	24.5
7 to <14		311	26.7
14 to <28		257	22
28 to <56		160	13.7
56 to 250		49	4.2
Days in hospital from ICU admission	1,166		
<7		63	5.4
7 to <14		200	17.2
14 to <28		310	26.6
28 to <56		224	19.2
56 to <250		126	10.8

Median age was 64 years (range 18 to 101, IQR 51 to 71): 62% were male, 81% were Caucasian and 13% Mediterranean, and 509 patients were hospitalised for more than 24 hours prior to ICU admission. Of these the mean time from hospital admission to ICU admission was 1.04 days. Median length of stay in the ICU was 11 days (IQR 6 to 23); median length of stay in hospital was 22 days (IQR 13 to 40) (Table 1).

A total of 718 (62%) patients had one or more co-morbid conditions, with cardiac and respiratory disease affecting 458 and 531 patients, respectively. Chronic obstructive pulmonary disease was documented in 278 (23.8%) patients, diabetes mellitus in 19.6% and a chronic neurological condition in 13.1%.

On the day of admission 884 (76%) patients required mechanical ventilation, with the number increasing to 962 patients (84%) within the first week of admission (Table 2). Median duration of mechanical ventilation was 7 days: 573 patients (49%) satisfied the criteria for septic shock. The median duration of inotrope/vasopressor support was 3 days. Renal replacement therapy was required during the first week of admission in 10% of patients. Median SOFA score on admission was 6 (IQR 4 to 9) and median APACHE II score was 20 (IQR 15 to 25).

**Table 2:** Severity scoring, physiological variables and changes on chest radiographs

**Clinical parameters**	**Number analysed**	**Median or number**	**IQR or %**
Scoring			
APACHE II score	1,166	20	15 to 25
SOFA II score	1,158	6	4 to 9
IDSA/ATS criteria for severe CAP	1,161	1,140	97.8%
Physiology			
Highest temperature, °C	1,160	38.0	37.2 to 38.6
Lowest temperature, °C	1,160	36.4	35.8 to 37
Lowest SBP, mmHg	1,161	91	80 to 105
Lowest MAP, mmHg	1,156	64	56 to 72
Highest heart rate. bpm	1,161	118	101 to 130
Lowest heart rate. bpm	1,161	84	70 to 95
Highest WCC, ×10^9^/L	1,160	14.2	9.9 to 20.7
Lowest WCC, ×10^9^/L	1,160	12.5	8.3 to 18
Lowest platelet count, ×10^9^/L	1,160	197	142 to 266
Haematocrit, %	1089	34.7	30 to 39
Bicarbonate, mmol/L	1049	22	19 to 26
Lowest sodium, mmol/L	1,159	136	132 to 139
Lowest potassium, mEq/L	1,159	3.7	3.4 to 4.2
PCO_2_, mmHg	1,144	24	5.5 to 40
pH	1,150	7.33	7.24 to 7.42
Serum bilirubin, μmol/L	1,135	13	8 to 20
Urea, μmol/L	1,068	11	6.8 to 20
Urine volume, cL/day	1,153	155	90 to 236.5
Creatinine, μmol/L	1,161	100	72 to 157
Respiratory rate, bpm	1,154	27	18 to 35
P:F ratio, mmHg	1,150	132	86 to 200
Renal failure during week 1 in ICU	1,161	411	37.1
Need for RRT during week 1 in ICU	1,161	189	17.2
Mechanical ventilation during week 1 in ICU	1,161	962	85.0
Septic shock on admission	1,161	573	49.4
Admission CXR changes	1166		
Lobar pattern on CXR		507	43.7
Localised pattern on CXR		290	25
Diffuse bilateral changes on CXR		340	29.3

All measurements recorded on day 1 of admission to the ICU unless stated otherwise. APACHE, acute physiology and chronic health evaluation; P:F ratio, ratio of the partial pressure to fractional inspired concentration of oxygen; SOFA. sequential organ failure assessment; IDSA/ATS, Infectious Diseases Society of America/American Thoracic Society; SBP, systolic blood pressure; MAP, mean arterial pressure; WCC, white cell count; CXR, chest radiograph.

Chest radiograph appearances were recorded as lobar consolidation in 43.7%, patchy localised consolidation in 25.0% and diffuse, bilateral changes in 29.3%.

**Figure 1:**
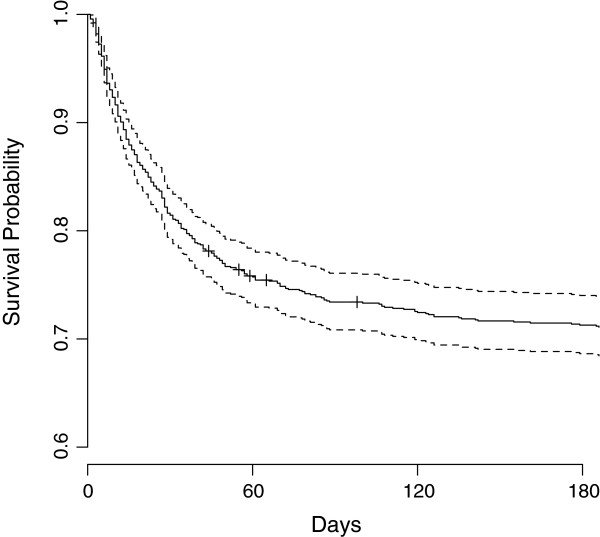
Kaplan-Meier curve demonstrating survival probability over a 6-month period for patients admitted to ICU with community-acquired pneumonia (CAP).

A total of 316 (27.3%) patients had died within 6 months of enrolment, 222 (19.0%) in the ICU, 63 in hospital after ICU discharge and 31 between hospital discharge and 6 months following ICU admission. The in-hospital mortality rate was 24.4% and the 28-day mortality rate was 17.3% (see Table 3 and Figure 1). Mortality was higher in mechanically ventilated patients, (24.9% at 28 days and 33.6% at 6 months) and in patients with septic shock receiving haemodynamic support with inotropes or vasopressors (28.8% and 38.6% respectively). The standardised mortality ratio derived from the APACHE II score was 0.69 (95% CI 0.68, 0.70) [28].

**Table 3:** Mortality rates in a cohort of 1,166 patients with community acquired pneumonia admitted to adult ICUs across Europe

**Time**	**Mortality status**	**Number**	**%**
6 months	Alive	848	72.7
	Dead	316	27.3
28 days	Alive	964	82.7
	Dead	202	17.3
Hospital	Alive	881	75.6
	Dead	285	24.4
ICU	Alive	944	81.0
	Dead	222	19.0

No causative organism was identified in over a third of patients (Table 4). *Streptococcus pneumoniae* (*Pneumococcus*) was the most commonly isolated organism (29% of cases), Positive microbiology was obtained from lung secretions/lavage in 47% of cases; blood culture in 22%; urinary antigen testing in 17%; blood serology in 10%; pleural fluid in 2.3% and other methods in 2%. Bacteraemia was present in 241 (22%) of patients and pleural infection/empyema in 32 (3%). *Pneumococcus* was the commonest organism causing bacteraemia and empyema, accounting for 119 episodes (49%) and 25 episodes (60%) respectively. Atypical organisms and viruses were rarely identified.

**Table 4:** Microbiological isolates from the GenOSept cohort (n = 1,166)

**Microbiology (organism)**	**Number**	**%**
*Streptococcus pneumoniae*	333	28.6
*Staphylococcus aureus*	69	5.9
*Legionella pneumophilia*	65	5.5
*Haemophilus influenzae*	56	4.8
*Pseudomonas aeroginosa*	52	4.5
*Klebsiella* spp.	27	2.3
*Chlamydia psittaci*	10	0.9
*Mycoplasma pneumoniae*	10	0.9
Mixed	64	5.5
Viral	16	1.4
Other	144	12.4
Unknown	427	36.6

Other organisms included: Candida spp. 30 (2.6%), *Escherichia coli* 15 (1.3%), *Streptococcus* spp.12 (1.0%), *Enterobacter* spp. 10 (0.9%), *Serratia Marcesens* 8 (0.7%).

### Univariate analysis

Isolation of *Staphylococcus aureus*, presence of septic shock, need for mechanical ventilation and renal replacement therapy all showed a strong association with outcome (Table 5).

**Table 5:** Univariate Cox proportional hazards models for 28-day and 6-month mortality for patients admitted to the ICU with CAP

	**Increment**	**28-day mortality**			**6-month mortality**		
		**Hazard ratio**	**95% CI**	***P*-value**	**Hazard ratio**	**95% CI**	***P*-value**
APACHE II score		1.1	1.08, 1.12	<1E-14	1.08	1.07-1.1	0.00E + 00
Staphylococcus aureus		1.65	0.99, 2.75	5.59E-02	2.03	1.38, 2.97	3.02E-04
Haematocrit	%	0.95	0.93, 0.98	2.98E-05	0.96	0.94, 0.98	6.19E-06
pH value	0.1 unit	0.7	0.62, 0.77	2.31E-11	0.75	0.69, 0.82	1.81E-10
Septic shock		1.98	1.48, 2.64	3.61E-06	1.6	1.28, 2	3.92E-05
Diffuse bilateral change on CXR		1.27	0.94, 1.7	1.14E-01	1.54	1.22, 1.94	2.44E-04
Bilirubin	10 μmol/L	1.04	1.02, 1.07	8.64E-04	1.04	1.02, 1.07	2.86E-04
Creatinine day 1	10 μmol/L	1.02	1.01, 1.03	1.41E-03	1.02	1.01, 1.03	1.85E-04
Lowest heart rate day 1		1.01	1, 1.02	2.35E-03	1.01	1.01, 1.02	4.62E-04
Lowest MAP day 1		0.97	0.96, 0.98	4.96E-06	0.98	0.97, 0.99	1.95E-06
Lowest SBP day 1	mmHg	0.98	0.97, 0.99	7.09E-08	0.98	0.98, 0.99	5.33E-07
Need for any ventilatory support		3.17	1.72, 5.82	2.01E-04	3.63	2.2, 6	5.07E-07
Mechanical ventilation		2.17	1.46, 3.21	1.19E-04	2.21	1.62, 3.03	6.41E-07
Renal failure in week 1		1.99	1.5, 2.64	2.04E-06	1.65	1.32, 2.07	1.40E-05
Need for renal replacement week 1		2.45	1.8, 3.32	9.00E-09	2.2	1.71, 2.82	6.09E-10
SOFAday1		1.12	1.08, 1.17	2.14E-08	1.11	1.08, 1.15	3.22E-10
Urine volume day 1	100 mL/day	0.97	0.96, 0.99	1.09E-04	0.97	0.96, 0.98	3.90E-06

All values are significant at a type I error rate of 5% after a Bonferroni correction to take account of the multiple testing of 64 variables (a *P*-value <0.00078 was considered to be statistically significant). Results are adjusted for age and sex. All day-1 variables unless specified otherwise. RRT, renal replacement therapy; MV, mechanical ventilation; NIV, non-invasive ventilation; SBP, systolic blood pressure; MAP, mean arterial pressure.

### Multivariate analysis

The factors most strongly associated with 28-day mortality were the APACHE II score calculated on day 1 (HR = 1.03, CI 1.01, 1.05) for each 1.0-point increase in score; haematocrit (HR = 0.97, CI 0.95, 0.99) for each 1% absolute increase; pH (HR = 0.83, CI 0.72, 0.95) for each 0.1-point increase and the need for ventilator support (HR = 2.29, CI 1.11, 4.72). Several other factors were associated with 6-month outcome. APACHE II remained significant (HR = 1.03, CI 1.01, 1.05) for each point increase, as did haematocrit (HR = 0.97, CI 0.95, 0.98) for each 1% increase. pH was strongly associated with 6-month outcome (HR = 0.86, CI 0.77, 0.96) for each 0.1 unit increase. Urine output on the first day was also associated with 6-month outcome (HR = 0.98, CI 0.97, 0.99) for each 100-mL increase in urine volume. Less strongly associated with outcome were bilirubin (HR = 1.004, CI 1.001, 1.006) and the lowest measured heart rate (HR = 1.01, CI 1.00, 1.02) for each 1-beat increase. Of the categorical variables, only diffuse bilateral changes on chest radiography (HR = 1.44, CI 1.11, 1.78) and the need for ventilator support (HR = 3.04, CI 1.64, 5.62) were significantly associated with death (Table 6).

**Table 6:** Multivariate Cox proportional hazards model for 28-day and 6-month survival for patients admitted to the ICU with CAP

	**Increment**	**Hazard**	**95% CI**	***P-*** **value**
**28-day mortality**				
APACHE II score		1.06	1.03, 1.09	4.23E-05
Mechanical ventilation		2.29	1.11, 4.72	2.56E-02
Haematocrit	1%	0.97	0.95, 0.99	6.61E-03
pH value	0.1 unit	0.83	0.73, 0.95	7.52E-03
**6-month mortality**				
APACHE II score		1.04	1.02, 1.06	5.67E-05
Mechanical ventilation		2.68	1.49, 4.85	1.06E-03
Urine volume day 1	100 mL/day	0.98	0.96, 0.99	1.28E-04
Lowest heart rate day 1		1.01	1.0, 1.01	4.60E-02
Haematocrit	%	0.97	0.95, 0.99	4.78E-04
Diffuse bilateral CXR		1.38	1.07, 1.78	1.32E-02

Final model is selected using a stepwise regression by Akaike-information-criteria. Results are adjusted for sex. Age correction included in APACHE II score. APACHE, acute physiology and chronic health evaluation; CXR, chest radiography.

## Discussion

This large, prospective study of 1,166 patients from 17 different countries provides a contemporary view of patients with severe CAP admitted to ICUs across Europe. The mortality rates were 17% at 28 days, 19% at intensive care discharge, 24% at hospital discharge and 27% at 6 months, giving a standardised mortality ratio based on the APACHE II scores of 0.69 (95% CI 0.68, 0.70).

A number of studies have documented mortality rates for patients with CAP admitted to ICU [5-7,9-22]. The considerable heterogeneity in admission policies, study design, guidelines compliance [21], and severity scoring in these studies probably accounts for the wide range of reported mortality rates and makes meaningful comparisons difficult. Most of these studies have used ICU admission rather than severity scores to indicate severe disease. Only three studies [12,18,21] defined censor points for death, which is important as in-hospital mortality increases following ICU discharge by between 15 and 27% [33]. Admission practices in different countries may also lead to large ranges in mortality. Take for instance one study of 395 patients admitted to a Spanish respiratory ICU in the 1990s the mortality rate was 5% but with rates of mechanical ventilation and septic shock of 9% and 2% respectively [14], whereas in a UK study published in 1997 the mortality rate was 58%, with mechanical ventilation and septic shock rates of 96% and 16% respectively [11]. The mortality rates reported here are more in keeping with other recent, large cohorts. The CAPUCI group analysed 529 patients admitted to over 30 Spanish ICUs between 2000 and 2002 and found ICU mortality rates of 28% with associated APACHE II scores of 19 [21]. They included both immunocompetent and immunosuppressed patients. In the 459 immunocompetent individuals the rate of death at ICU discharge was slightly lower at 25%, a figure that is closer to the 19% seen in the GenOSept cohort, (in which immunocompromised patients were excluded). Some studies have examined larger cohorts of patients admitted to hospital with CAP of varying severity, but have reported outcomes for the smaller subgroup of patients admitted to the ICU. The GenIMS investigators, for example examined a population of 1,895 CAP patients; 302 were admitted to the ICU, 52 (17.3%) of whom died during their hospital stay [3]. In the PORT study, 170 of 1,339 patients were admitted to the ICU with an in-hospital mortality rate of 23.3% [24].

The standard censor point for most interventional ICU studies is 28 days, although it is recognised that there is a significant attrition rate post ICU discharge. It is also well-recognised that in CAP patients there is an increased death rate in the months following discharge [34], and in patients with sepsis there is significant excess mortality for at least five years [35]. In one study, the death rate of ICU patients between 28 days and 6 months was 9% in patients with sepsis, similar to the 8% seen in ICU patients without sepsis [36] and the 10% found in the present study. This compares to an increase in mortality from 18.2% at 30 days to 24.8% at 90 days in the subgroup of ICU patients in the PORT study [24] and an increase from 17.3% to 34.8% at 12 months in the GenIMS cohort of ICU patients [3].

Although the microbiological methodology was not standardised, our findings are consistent with previous studies of severe CAP. Notably, *Streptococcus pneumoniae* accounted for 28% of all cases and no aetiological agent was identified in over a third [37]. Within the GenOSept cohort the rate of *Streptococcus pneumoniae* was higher than previously reported and was mirrored by a decrease in the number of cases where no aetiological agent was identified, suggesting that detection rates may have improved, rather than there being a true increase in incidence. Pneumococcal antigen testing in urine and other bodily fluids has become the standard of care in many institutions and has good positive and negative predictive value both in hospitalised CAP patients [38] and in those admitted to ICU [39]. Amongst the 333 patients with confirmed pneumococcal pneumonia in the present study, a total of 429 positive results were obtained. Of these 123 (29%) were positive urinary antigen tests and 42 (10%) were based on positive blood serology. Further evidence to support this apparent increase in detection rates is provided by a recent study that reported detection rates of 50% for *Streptococcus pneumoniae*, with 16% of cases being diagnosed using antigen testing [21]. In comparison, the BTS study from 1992 reported a rate of *Streptococcus pneumoniae* infection of 18%, with positive antigen testing in only 6% of patients [7,21].

In hospitalised patients with CAP, as many as 18 to 29% may involve a viral infection, the virus being the only organism isolated in 10% [40]. Failure to respond to standard antimicrobial therapy means that more patients with viral pneumonia are likely to be admitted to ICU. Certainly patients with co-morbidities have a higher incidence of viral infection. In the present study viral infections were rarely identified. This raises the question as to whether a more aggressive search for viral pathogens should be conducted in all ventilated patients, coupled with more frequent and targeted use of antiviral therapy.

Multivariate analysis identified four variables (APACHE II score, haematocrit, mechanical ventilation and pH) that were independently associated with outcome at both 28 days and 6 months The need for mechanical ventilation was related to a worse outcome at both 28 days and 6 months, consistent with other data showing respiratory failure to be an independent predictor of mortality in many categories of critically ill patients [28,29]. The persistence of this relationship for up to 6 months reflects the fact that many patients who have received ventilator support will continue to have significant neuromuscular weakness and be at risk for a prolonged period after discharge from the ICU [41]. It may be that over-aggressive use of intravenous fluids, reflected in a dilutional reduction in haematocrit worsens lung injury and thus prolongs the need for mechanical ventilation. Conservative fluid management has been associated with better outcomes, albeit in the later phases of critical illness [42]. A key element of early goal-directed therapy in patients with sepsis is blood transfusion to maintain the haematocrit, perhaps accounting for the positive association between better outcomes and a higher haematocrit in the present, and other studies [43]. Similarly the positive independent relationship between a higher pH on admission and a better outcome may reflect more effective early resuscitation.

Although there was no association between the admission P:F ratio and outcome, diffuse bilateral changes on the chest radiograph (suggesting a diagnosis of acute lung injury/acute respiratory distress syndrome) independently predicted a worse outcome at 6 months. This is consistent with other studies showing a mortality rate for ARDS much higher than that seen in our cohort of patients with CAP [41].

Urine volume, renal failure and the need for renal replacement therapy were associated with worse outcome in the univariate analysis and there was a clear independent association between urine output and mortality at 6 months. Acute kidney injury (AKI) has been shown to be independently associated with higher ICU and in hospital mortality rates [44,45]. The need for ongoing renal support in those with AKI is estimated to be 14%, perhaps explaining the association with outcome at 6 months.

Other studies have attempted to determine independent risk factors for death from CAP. For example, the presence of septic shock has been associated with odds ratios for risk of death ranging from 2 to 141 but inevitably with wide confidence intervals due to the small numbers of patients included in these analyses [5,6,15,16,22]; also the lack of a consistent censor point complicates interpretation of these findings. We found an association between septic shock and outcome on univariate analysis but this effect was not seen in multivariate analysis, perhaps reflecting improvements in the acute management of shock.

This study has two important limitations. Firstly participating centres were at liberty to decide which patients they would enrol; subjects were not, therefore, enrolled consecutively, thereby introducing a potential for selection bias. Also there was considerable variation in the number of patients recruited in each country and some centres contributed only small numbers of patients. Nevertheless there was a wide range of ages, severity of physiological derangement and co-morbidities, whereas APACHE II scores and ventilation rates were similar to previous studies, suggesting that a significant, systematic selection bias is unlikely. Secondly, microbiological protocols were not standardised. On the other hand our observations therefore reflect current approaches to microbiological diagnosis in routine clinical practice across Europe.

## Conclusion

The ICU mortality rate in this contemporary cohort of patients admitted to ICUs across Europe with severe CAP was 19%, rising to 24% at hospital discharge and 27% at 6 months. *Streptococcus pneumoniae* was the commonest cause of CAP, but in many cases the infecting organism was not identified. The need for ventilator support, and the presence of diffuse bilateral infiltrates on the chest radiograph, as well as lower haematocrit, urine volume and pH on admission to ICU were independent predictors of a worse outcome.

## Key messages

• The mortality rate from severe CAP in patients admitted to ICU is 27% at six months

• *Streptococcus pneumonia* remains the most commonly isolated organism

• No microbiological diagnosis is found in a third of patients

• The need for mechanical ventilation is a strong predictor of a poor outcome

• pH, haematocrit, urine output and diffuse changes on chest radiography all predict a worse outcome

## Abbreviations

APACHE II: acute physiology and chronic health evaluation II score; BTS: British Thoracic Society; CAP: community-acquired pneumonia; CAPUCI: community-acquired pneumonia in intensive care study; ECCRN: European Critical Care Research Network; eCRF: electronic case report form; ESICM: European Society of Intensive Care Medicine; HDU: high dependency unit; ICNARC: Intensive Care National Audit Research Centre; IDSA/ATS: Infectious Diseases Society of America/American Thoracic Society; MAP: Mean arterial blood pressure; NIV: non-invasive ventilation; P:F: ratio of partial pressure to fractional concentration of oxygen; SBP: systolic blood pressure; SOFA: sequential organ failure assessment score; WCC: white cell count.

## Competing interests

Paula Hutton was part-funded by the National Institute for Health Research Clinical Research Network. Andrew P Walden has no conflict of interest; Geraldine M Clarke has no conflict of interest; Stuart McKechnie has no conflict of interest; Paula Hutton has no conflict of interest; Anthony C Gordon has no conflict of interest; Jordi Rello has no conflict of interest; Jean-Daniel Chiche has no conflict of interest; Frank Stueber has no conflict of interest; Chris S Garrard has no conflict of interest; Charles J Hinds has no conflict of interest.

## Authors’ contributions

APW prepared the database for analysis, prepared the first copy of the manuscript and coordinated all manuscript revisions; GMC performed the primary statistical analysis, helped in the writing of the manuscript and provided the tables and figures; SMcK assisted in preparation of the database for analysis and helped in writing and reviewing the manuscript; PH helped with the preparation of the database for analysis and helped in writing and reviewing the manuscript; ACG helped in the design of the GenOSept project and in the writing an reviewing of the manuscript; JR helped in the design of the GenOSept project and in writing and reviewing the manuscript; J-DC helped in the design of the GenOSept project and in writing and reviewing the manuscript; FS helped in the design of the GenOSept project and in writing and reviewing the manuscript; CSG helped in the design of the GenOSept project and in writing and reviewing the manuscript; CJH helped in the design of the GenOSept project and in writing and reviewing the manuscript. All authors read and approved the final manuscript.

## Supplementary Material

Additional file 1 List of variables included in univariate analysis. Click here for file
