# Supplementary material for: Patients with community acquired pneumonia admitted to European intensive care units: an epidemiological survey of the GenOSept cohort
Source: Crit Care. 2014 Apr 1;18(2):R58. doi: 10.1186/cc13812 (PMC4056764; doi:10.1186/cc13812)
Supplement: Additional file 1 — List of variables included in univariate analysis. [file cc13812-S1.rtf]

Additional file 1

List of Variables included in Univariate Analysis


Demographics

Age

Sex

APACHE II score (day 1)

SOFA score (day 1)


Organism type

Streptococcus pneumoniae

Haemophilus influenzae

Staphylococcus aureus

Pseudomonas aeruginosa

Legionella pneumophila

Mixed organism

Unknown

Viral


Culture type

Lung secretions

Blood culture

Pleural effusion

Serology

Other

Complicating factor

Pleural effusion

Empyema

Recent hospital discharge

Home oxygen prior to admission

Comorbidities

Cardiovascular disease

Respiratory disease

2


Neurological disease

Renal disease

Underlying malignancy

Diabetes mellitus

History of previous serious infection

History of severe exercise limitation


Physiological parameters (day 1)

Temperature

Mean arterial pressure

Heart rate

Urine volume

Respiratory rate

P:F ratio

Presence of septic shock.

Presence of renal failure

Need for organ support

Need for ventilatory support

Need for invasive mechanical ventilation

Renal replacement therapy (in first week)

Laboratory measurements (day 1)

Creatinine

White cell count

Platelets

Whole blood haematocrit

Serum sodium

Serum potassium pH value
Partial pressure of CO2

Serum bicarbonate

Serum bilirubin

Serum urea

Radiological changes

Lobar changes on CXR

3


Localised changes on CXR

Diffuse bilateral changes on CXR
4


Defintion of Immunocompromise


known regular systemic corticosteroid therapy, exceeding 7mg/kg/day of hydrocortisone or equivalent, within three months of admission and prior to acute episode, known regular therapy with other immunosuppressive agents, e.g. azathioprine, known to be HIV positive or have acquired immunodeficiency syndrome as defined by the Centre for Disease Control, neutrophil count less than 1000 mm-3 due to any cause including metastatic disease and haematological malignancies or chemotherapy, but excluding severe sepsis; organ or bone marrow transplant receiving immuno-suppressive therapy.


Non-proportionality of hazard in the Cox regression analysis


Hazard ratios for lowest MAP, urine volume, renal failure, renal replacement therapy and pH value showed evidence of non proportionality of hazards (P value < 0.01). Figure 3 shows estimated non-proportional time dependent hazard ratios for these variables. Every 1 mmHg increase in MAP on day 1 confers a protective effect on 28-day mortality of approximately 8% on day 1 reducing steadily to no effect on approximately day 15. The protective effect of a 100mL/day increase in urine volume (reduced hazard by approximately 12% on day 1 decreases to no effect on approximately day 15) is apparent only for the first two weeks and shows no effect thereafter. The protective effect of a 0.1 increase in pH value on day 1 decreases rapidly over the first 15 or so days and then levels off but maintains a small effect for the remaining 13 days. The hazard for individuals with renal failure compared to individuals without renal failure is approximately 10 fold on day 1 but reduces steadily to no difference on approximately day 12. Similarly, the hazard comparing patients who do and who do not undergo renal replacement therapy in the ICU is at its greatest on day 1 indicating a 15 fold increase in risk but reducing steadily to no risk at approximately day 15. Finally, the hazard comparing individuals with and without shock on admission is approximately 5 on day 1 but decreases steadily to no difference over the 28 days.

5


Figure 1 Estimated hazard ratios (solid lines) with 95% confidence intervals (dashed lines) for variables showing evidence of nonproportionality of hazard in a univariate Cox proportional hazards regression for 28-day survival: (A) urine volume (mmHg); (B) renal failure; (C) renal replacement therapy; (D) PH value. The horizontal dotted line shows the estimated fixed hazard ratio.

Figure 2  Estimated hazard ratios (solid lines) with 95% confidence intervals (dashed lines) for variables showing evidence of nonproportionality of hazard in a univariate Cox proportional hazards regression for 6-month survival: (A) Renal Failure and; (B) 0.1 PH value. Spline smooth estimates of time dependent hazard ratios with pointwise confidence bands were calculated using Schoenfeld residuals The horizontal dotted line shows the estimated fixed hazard ratio.  

6

Figure 1


7


Figure 2

8


Database and Quality Control


The case-report form was developed and tested by CH, AG, CG, JDC and JM, together with other members of the GenOSept Consortium. A specific electronic case report form (eCRF) including alerts and audit trail of data entry, was developed by Lincoln©, Paris, France using software developed in collaboration with JDC. The Quality Assurance team (P.H., P.P., C.G. A.S., A.G. and C.H.) systematically reviewed all data. Data queries (DQs) were created and flagged within the eCRF for missing or erroneous data. Local investigators were notified of the DQs by email and received guidance notes when necessary. Up to the end of December 2009, a total of 9115 valid DQs were created; 90% of these DQs were responded to by investigators. Common errors included entering data using non-standard units and incomplete data entry. All patients' eCRFs were adjudicated by experienced intensive care physicians (C.H., C.G. and A.G.) for diagnosis of CAP and sepsis. Clarification or confirmation was sought from investigators if there was any uncertainty about the patient's study eligibility and a final decision then made regarding the inclusion of individual patients. Regular quality assurance reports were sent to the GenOSept Management Committee for review and National Investigators were contacted regarding quality issues when necessary.


List of Study Personnel Responsible for Database Development and Quality Control.


C.H. – Charles Hinds, Professor of Intensive Care Medicine, William Harvey Research Institute, Barts and The London Queen Mary School of Medicine C.G. – Dr Chris Garrard, Consultant in Intensive Care Medicine, John Radcliffe Hospital, Oxford.

J-D.C. – Professor Jean-Daniel Chiche, Professor of Intensive Care Medicine, Cochin hospital, France.

J.M – Dr Julian Millo, Consultant in Intensive Care Medicine, John Radcliffe hospital, Oxford.

9


A.G. – Dr Anthony Gordon, Clinical Senior Lecturer and Consultant in Critical Care Medicine, Imperial College London.

P.H. – Ms Paula Hutton, chief research nurse, John Radcliffe Hospital, Oxford P.P. – Ms Penelope Parsons, research nurse, John Radcliffe hospital, Oxford. A.S. Ms Alex Smith, research nurse, John Radcliffe hospital, Oxford.


List of Contritbuting Centres and Investigators


Belgium:

Intensive Care Unit, AZ-VUB university hospital, 101 Laarbeeklaan, Brussels; Intensive Care Unit, Chu Charleroi, 92 Boulevard Janson, Charleroi; Soins Intensifs, Clinique Saint Pierre, 9 Avenue Reine Fabiola, Ottignies; Intensive Care, Cliniques Universitaires Saint Luc (UCL), 10 Avenue Hippocrate, Brussels; Intensive Care, University hospital, 185 De Pintelaan, Gent; Soins Intensifs, Cliniques de l'europe - St Michel,150 Rue de Linthout, Brussels.


Croatia:

Medic, Emergency and Intensive Care Medicine/Internal Medicine, Clinical hospital Rebro, 12 Kispaticeva, ZagrebAnestesiology and ICU, Clinical Hospital Rebro, 12 Kispaticeva, Zagreb.


Czech Republic:

Anesteziologicko-reuscitacni klinika, Fakultní Nemocnice u Svaté Anny, 53

Pekaøská, Brno; Anesteziologicko-resuscitacni oddeleni, Fakultni Nemocnice Brno, 20 Jihlavská, Brno-Bohunice; Klinika anestezie, resuscitace a intenzivni mediciny, Fakultni Nemocnice Hradec Kralove, 581 Sokolská, Hradec Kralove; Chirurgicka klinika, Fakultní Nemocnice s Poliklinikou Ostrava, 1790 listopadu, Ostrava-Poruba; Anesteziologicko-resuscitacni klinika, Fakultni Nemocnice Plzen, 80 Alej Svobody Plzen; Anestezie, resuscitace a intenzivni medicina, Masarykova Nemocnice,

3316/12A Sociální péèe, Ústi Nad Labem; Anesteziologicko-resuscitacni oddeleni, Nemocnice Znojmo, 11 Janského, Znojmo; Anesteziologicko-resuscitacni oddeleni, Krajska Nemocnice Liberec, 10 Husova, Liberec.

10


Estonia:

General ICU, Tartu University Hospital, 1a L. Puusepa, Tartu; Pulmonary ICU, Tartu University Hospital,1a L. Puusepa, Tartu.


France:

Service de Réanimation Médicale, Hopital Cochin, 27 rue du Fbg St Jacques, Paris; Service de Réanimation Médicale, HEGP, 20 rue Leblanc, Paris; Service de Réanimation Médicale, Hotel Dieu, 1 place du Parvis Notre Dame, Paris; Service de Réanimation Médicale, Saint Joseph, 185 rue Raymond Losserand, Paris; Service de Réanimation Médicale, Chru Angers, 4 rue Larrey, Angers; Service de Réanimation Médicale, Chu de Nice, Rte St Antoine Ginestière, Nice; Service de Réanimation Médicale, Chu Purpan, Chu Toulouse- Hôpital Purpan, Toulouse; Service de Réanimation Médicale, Ch Versailles, 177 rue de Versailles, Le Chesnay.


Germany:

Klinik für Herzchirurgie, Klinikum der Stadt Ludwigshafen am Rhein GGMBH, 79 Bremserstraße, Ludwigshafen; Klinik und Poliklinik für Anästhesiologie und Intensivmedizin, Klinikum Greifswald, 23b Friedrich-Loeffler-Straße, Greifswald; Klinik fur Anästhesiologie und operative Intensivmedizin, Klinikum Augsburg, 2 Stenglinstr., Augsburg; Klinik und Poliklinik für Anaesthesiologie und Intensivtherapie, Universitätsklinikum Dresden, 74 Fetscherstrasse, Dresden; Klinik für Anästhesiologie und Intensivtherapie, Klinikum der Friedrich Schiller Universität, 101 Erlanger Allee, Jena; Klinik für Anästhesie und Intensivmedizin, Westküstenklinikum Heide, 50 Esmarchstraße, Heide; Abt. fur Anästhesiologie und Intensivtherapie, Fachkrankenhaus Coswig - centre for pneumology and thoracic surgery, 21 Neucoswiger Str., Coswig; Klinikum der Medizinischen Fakultät der Martin Luther Universität Halle-Wittenberg, 40 Ernst-Grube-Str., Halle; Klinik für Intensivmedizin, University medical center Eppendorf, 52 Martinistr., Hamburg; Klinik und Poliklinik für Anästhesiologie und Operative Intensivmedizin (Turmgebäude 2OG Zimmer 221), Universitätsklinikum Bonn, 25 Sigmund-Freud-Str., Bonn; Internal Medicine, Universitätsklinikum Mainz, 1 Langenbeckstrasse, Mainz.

11


Greece:

Intensive Care, Sismanoglion general hospital, Marousi, Athens; Critical care, Attikon university hospital, 1 Rimini, Xaidari.


Hungary:

Surgery 1St, Semmelweis University, 78 Ulloi Ut, Budapest


Eire:

Intensive care unit, St James hospital, James Street, Dublin; Intensive care unit,

Adelaide Meath and national children's hospital, Tallaght, Dublin; Anaesthesia and

Intensive care, National university hospital Galway, Newcastle Road, Galway; Anaesthesia & Intensive Care Medicine, James Connolly memorial hospital, Blanchardstown, Dublin; Department of Anaesthesia and Intensive Care Medicine, Cork university hospital, Wilton, Cork.


Israel:

Carmel medical center, Haifa; General Intensive care unit, Haemek medical center, Afula; Anaesthesiology and critical care medicine, Hadassah medical center, Kiryat Hadassah, P.O. Box 12000, Jerusalem


Italy:

Anestesiologia e Rianimazione 3, Ospedale S. Giovanni Battista – Molinette, 88 Corso Bramante, Torino; Anestesia e Rianimazione, Ospedale S.Giovanni Bosco, 3 Piazza Donatori del Sangue, Torino; Dr Rianimazione SOD 2, AOU Careggi, 85 Viale Morgagni, Firenze; Anestesia e Rianimazione, Ospedale Maggiore, 35 Via Francesco Sforza, Milano; Terapia Intensiva, Universita Degli Studi Milano Bicocca A.O. San Gerardo, 106 Via Donizetti, Monza; Anestesia e Rianimazione, Ospedale S.Orsola Malpighi, 9 Via Massarenti, Bologna; Anestesia e Rianimazione, Ospedale S.Giovanni Addolorata, 8 Via dell'Amba Aradam, Roma; Scienze Anestesiologische, Medicina Critica e Terapia del Dolore, Policlinico Umberto I, 155 Viale del Policlinico, Roma.


Netherlands:

Intensive care unit, Erasmus medical centre, 230 Gravendijkwal, Rotterdam.

12


Poland:

Anaesthesiology and Intensive Therapy, Medical university, 7 Debinki St, Gdansk; Klinika Anestezjologii i Intensywnej Terapii sp Centralny Szpital Kliniczny Sam; Military teaching hospital; Szpital Wojewodzki/regional hospital; University hospital n°2; Szpital Wojewodzki; University hospital of Bydgoszcz; Wroclaw medical University.


Serbia:

Military medical academy; Clinical center Kragujevac.


Spain:

University hospital de Bellvitge; hospital Universitario Puerta del Mar; hospital Universitario de Gran Canaria; hospital de la Princesa; hospital Nostra Senyora de Meritxell; hospital de Mataro; hospital clinico San Carlos; hospital Universitari de Terragona Joan XXIII; hospital Sagunt; centro medico Delfos; hospital de Huesca; hospital general de Segovia; Basurto hospital; hospital Universitario Arnau de Vilanova; hospital general Yague; hospital Universitario Puerto Real; hospital Universitario de Girona ; hospital General de Vic; Hospital Verge De La Cinta.


United Kingdom:

Aberdeen Royal Infirmary; Addenbrooke's Hospital; Barts and the London NHS trust; Broomfield hospital; Charing Cross Hospital; Chelsea and Westminster Hospital; Cheltenham general hospital; Colchester General Hospital; Freeman Hospital; Frimley Park hospital; Hammersmith hospital; Homerton University hospital; Hope hospital; Huddersfield royal infirmary; Hull royal infirmary; Ipswich hospital NHS trust; John Radcliffe hospital; Leeds general infirmary; Leicester royal infirmary; Manor hospital, Walsall; Norfolk & Norwich NHS trust; Queen Elizabeth hospital, King's Lynn ; Queen Elizabeth University hospital; Royal Berkshire Hospital; Royal Hallamshire hospital; Royal Preston hospital; Royal Sussex county hospital; Royal Victoria infirmary; Southend hospital NHS trust; st James University hospital; the Great Western hospital; the James Cook University hospital; The Whittington hospital; UCLH Middlesex hospital; University hospital Lewisham; University

13


hospital of Wales; University hospital, Coventry; Worthing hospital; Wythenshawe

Hospital

14


National Co-ordinators:

Austria	H Novak
Belgium	P Damas

Croatia	V Gasparovic
Czech Republic	V Sramek

Estonia	S Sarapuu
France	J-D Chiche

Germany	F Bloos
Greece	A Armagandis

Hungary	I Bobek
Ireland	T Ryan

Israel	Y Weiss
Italy	P Cotogni

Netherlands	J Hazelzet
Poland	A Mikstacki

Serbia	M Surbatovic
Spain	G Sirgo

United Kingdom	C Hinds


Principal Investigators

Austria AT
H Novak

Belgium BE
H Spapen

P Biston
T Dugernier

P.F. Laterre
P Damas

V Collin

Croatia HR
M Grgic Medic

T Mahecic


Czech Republic CZ
V Sramek
J Mannova

D Bares
O Marek

I Satinsky
I Novak

M Panko
S Vojtech

I Zykova

15


Estonia EE
S Sarapuu

France FR
J D Chiche

J L Diehl
A	Rabbat 

B	Misset 
P	Asfar 

H	Hyvernat 

P	Sanchez J-P Bedos 


Germany DE
F Isgro
M Grundling

U Jaschinski
M Ragaller

F Bloos
S Schroder

J Krassler
A Nierhaus

C Putensen
M Weiss

Prof Larsen
M Lauterbach

Greece GR
D Evrenoglou
A Armaganidis

Hungary HU
K Darvas
I Okros

Ireland IE
T Ryan
M Donnelly

J Laffey
C Cody

C	Motherway 
D	Breen 


Israel IL
R Pizov

A Lev
Y Weiss

16


Italy IT
V. M Ranieri
S Livigni

P Pelaia
R Tufano

A.R De Gaudio
L	Gattinoni 

A	Pesenti 
M	Capuzzo 

G	Sangiorgi 
F	Turani 

F	Conforto 
F	Bilotta 

Netherlands NL
B Van Der Hoven

Poland PL
A	Siemiatkowski 

D	Maciejewski 
M	Wujtewicz 

E	Karpel 
A	Ziajka 

R	Gajdosz 
W	Gaszynski 

A	Nestorowic 
W	Kowalski 

A	Mikstacki 
L	Drobnik 

L	Krawczyk 
J	Jastrzebski 

A	Kanski 
W	Koscielniak 

M	Mikaszweska-Sokolewicz 
K	Kusza 

A	Kubler 
B	Jozef 

Serbia RS
M Surbatovic
J Jevdjic

Spain ES
X L Perez Fernandez
R l Sierra

J Sole-Violan
N Carrasco

A Margarit Ribas
J C Yebenes

17


A Valverde Conde
G Sirgo

E Gomez Martinez
F F Dorado

L	Labarta 
L	Cambra 

M	A Vidarte Ortiz 
M	B  Castello 

J	L Fernandez 
J	Gil Cebrian 

J	M Sirvent 
M	C Martin 

United Kingdom UK
C	Hinds 

C	Garrard 

A	Johnston 
D	Watson 

S	Baudouin 
M	Watters 

R	Venn 
J	Bion 

D	Higgins 
M	J. Garfield 

S	Pambakian 
J	Thompson 

J	Durcan 
A	Kapila 

G	Bellingan 
S	Fletcher 

A	Bentley 
A	Mallick 

R	Bailie 
I	Krupe 

M	Oram 
M	Hayes 

E	Wheatley 
S	Murdoch 

S	Bonner 
N	Webster 

G	Findlay 
M	Blunt 

G	Mills 
G	Thomas 

S	Drage 
A	Timmins 

S	Pesian 
A	Gordon 

M	Kuper 
P	Hall 

18


P Venkatesh
J Moreno Cuesta

S Laha
A Guleri

I Smith
A Krige

P Watt

Research Nurses/Fellows

E Svoren

A Purdy

E McLees

P Hutton

P Parsons

A Smith

R Farras-Arraya

C Higham

C Ryan
C Pirie

K Mayell

K Challis

S Morris

N Waterhouse
V Flitchett

J Margalef

Dr Mowatt
P Hudson

R	Gupta 

J	Wilde 

S	Lees 

A	Nillson 

S	Andrews 

E	Simpson 
S	Mappleback 

S Burfield

L Sherrard Smith
V Jamieson

K Williamson

E Thomson

S Rogers

N Wilson

S Bowrey

N Rich

N  Griffin-Teal

C Mitchell-Inwang

S Williams
K Swan

S Smolen

19


C Jones

H Prowse
N Jacques
J Atkinson

S Boluda

A Bakarr Karim

J Hyun Ryu
J Nagle

G Bercades
M Rosbergen

G	Glister 

F	Jefferies 

D	Downs 

K	Millward 

S	Elliot 

J	Thornton 

D	Mawer 

J	Calderwood 

I	Whitehead 

V	Goodridge 

K	Hugill 

K	Colling 

S	Roughton 

H	Tennant 

J Taylor

S Hall

J Addison

L Macchiovello
E Hutcheon

C Underwood

K Wong

J Collins

N Mills
E Calton

J Sorrell

S Lowes

L	Ortiz-Ruiz De Gordoa 

A	Ghosh 

O	Thunder 

N	Wheatley 

M	Templeton 

R	Wilson 

C	Gibbs 

L	Mountford 
J	Gonzalez-Moreno 

M	Ainsworth 

S	Pahary 

S Musaad
J Hewlett

20


J	England 
G	Ward 

S	Nyabadza 
S	Clay 

C	Gibson 
E	Archer 

K	Hotchkiss 
D	Gocher 

J	Daglish 
M	Dlamini 

J	Baldwin 
N	Doherty 

J	Cocker 
N	Waddington 

N	Smith 
D	Harrison 

M	Bland 
L	Bullock 

P Raymode
